# The PRT6 N‐degron pathway restricts VERNALIZATION 2 to endogenous hypoxic niches to modulate plant development

**DOI:** 10.1111/nph.16477

**Published:** 2020-03-16

**Authors:** Anne‐Marie Labandera, Hannah M. Tedds, Mark Bailey, Colleen Sprigg, Ross D. Etherington, Olunwatunmise Akintewe, Geetika Kalleechurn, Michael J. Holdsworth, Daniel J. Gibbs

**Affiliations:** ^1^ School of Biosciences University of Birmingham Edgbaston B15 2TT UK; ^2^ School of Biosciences University of Nottingham Loughborough LE12 5RD UK

**Keywords:** flowering time, hypoxia, N‐degron pathway, polycomb, PRC2, proteolysis, root, vernalization

## Abstract

- VERNALIZATION2 (VRN2), an angiosperm‐specific subunit of the polycomb repressive complex 2 (PRC2), is an oxygen (O_2_)‐regulated target of the PCO branch of the PRT6 N‐degron pathway of ubiquitin‐mediated proteolysis. How this post‐translational regulation coordinates VRN2 activity remains to be fully established.
- Here we use *Arabidopsis thaliana* ecotypes, mutants and transgenic lines to determine how control of VRN2 stability contributes to its functions during plant development.
- VRN2 localizes to endogenous hypoxic regions in aerial and root tissues. In the shoot apex, VRN2 differentially modulates flowering time dependent on photoperiod, whilst its presence in lateral root primordia and the root apical meristem negatively regulates root system architecture. Ectopic accumulation of VRN2 does not enhance its effects on flowering, but does potentiate its repressive effects on root growth. In late‐flowering vernalization‐dependent ecotypes, VRN2 is only active outside meristems when its proteolysis is inhibited in response to cold exposure, as its function requires concomitant cold‐triggered increases in other PRC2 subunits and cofactors.
- We conclude that the O_2_‐sensitive N‐degron of VRN2 has a dual function, confining VRN2 to meristems and primordia, where it has specific developmental roles, whilst also permitting broad accumulation outside of meristems in response to environmental cues, leading to other functions.

## Abstract 2

See also the Editorial on this article by Sasidharan et al., **229**: 5–7.

## Introduction

Plants need to accurately regulate gene expression to appropriately coordinate their development in response to changing environmental conditions. Such regulation occurs at the direct transcriptional level, through the action of transcription factors, but also epigenetically through a combination of DNA methylation and modification of histone tails in the nucleosomes that make up chromatin. One of the best characterized histone modifying enzymes in eukaryotes is the polycomb repressive complex 2 (PRC2), a multisubunit holoenzyme that catalyses the deposition of the Histone H3 lysine 27 trimethylation (H3K27me3) mark on chromatin (Simon & Kingston, 2009; Margueron & Reinberg, 2011). H3K27me3 promotes compaction of nucleosomes, and therefore acts as a repressor of transcription through switching off gene expression by limiting transcription factor occupancy. The canonical PRC2 comprises four core subunits, and several of these have expanded in number in plants, indicating enhanced flexibility in the composition and activity of individual PRC2s (Hennig & Derkacheva, 2009; Mozgova *et al.*, 2015). PRC2 complexes in *Arabidopsis thaliana* (hereafter Arabidopsis) are named according to which of three homologues of the *Drosophila melanogaster* SUPPRESSOR OF ZESTE 12 (Su(z)12) subunit they recruit: FERTILIZATION INDEPENDENT 2 (FIS2), EMBRYONIC FLOWERING 2 (EMF2) and VERNALIZATION 2 (VRN2). Both EMF2 and VRN2 are expressed in sporophytic tissues, where they form PRC2 complexes in association with the methlytransferases CURLY LEAF (CLF) or SWINGER (SWN). CLF and SWN can act interchangeably with EMF2 and VRN2 to regulate overlapping and distinct functions, particularly in the control of shoot development and flowering (Gendall *et al.*, 2001; Yoshida *et al.*, 2001). By contrast, the expression and activity of FIS2 and its cognate methyltransferase MEDEA (MED) are exclusive to the gametophyte, where FIS2‐PRC2 prevents fertilization in the absence of pollination (Yadegari *et al.*, 2000). Common to all three PRC2 complexes is FERTILIZATION INDEPENDENT ENDOSPERM (FIE), a homologue of Drosophila EXTRA SEX COMBS, and the only component in Arabidopsis that is not encoded by multiple family members (Ohad *et al.*, 1999).

VRN2 was first identified as a major regulator of the vernalization response in *Arabidopsis* ecotypes that require prolonged winter to initiate flowering in spring (Chandler *et al.*, 1996; Sheldon *et al.*, 2000; Gendall *et al.*, 2001). During cold exposure, VRN2‐PRC2 accumulates, and through associations with other cold‐specific accessory proteins – including the PHD protein VERNALIZATION INSENSTITIVE3 (VIN3) and its homologue VRN5 – it contributes to histone methylation and epigenetic repression of the floral inhibitor gene *FLOWERING LOCUS C (FLC)* (Sung & Amasino, 2004; Wood *et al.*, 2006; Greb *et al.*, 2007; Costa & Dean, 2019). PRC2‐mediated *FLC* silencing occurs in distinct phases; initial methylation at a nucleation site in the cold requires VRN2‐PRC2 in association with SWN, whilst subsequent H3K27me3 spreading throughout the *FLC* gene body occurs in the warmth and is dependent on PRC2‐CLF (Yang *et al.*, 2017; Costa & Dean, 2019). Mutations of VRN2 and VRN2‐like proteins in Arabidopsis*,* rice and *Medicago truncatula* (Medicago) also lead to nonvernalization‐associated flowering phenotypes, indicating additional roles for VRN2 in autonomous flowering pathways (Gendall *et al.*, 2001; Yang *et al.*, 2013; Jaudal *et al.*, 2016). Further to its role in vernalization, VRN2 has been implicated in the control of seed dormancy (Auge *et al.*, 2017), seed development (Roszak & Kohler, 2011), vascular patterning and root cell proliferation (de Lucas *et al.*, 2016), somatic cell de‐differentiation (Ikeuchi *et al.*, 2015), and hypoxia and submergence tolerance (Gibbs *et al.*, 2018).

Although many functions and targets of plant PRC2s are known, direct mechanisms controlling their activity, composition, and specificity are less well established. Spatiotemporal differences in the expression of SWN, CLF, EMF2 and VRN2 have been demonstrated in specific root cell types, indicating that the patterning of their expression contributes to their diverse functions (de Lucas *et al.*, 2016). However, discrepancies in promoter activity, mRNA abundance, and protein accumulation indicate that PRC2 subunits are also under post‐translational control (Wood *et al.*, 2006; de Lucas *et al.*, 2016). We previously identified VRN2 as an oxygen (O_2_)‐regulated target of the PCO branch of the PRT6 N‐degron pathway of proteolysis via its conserved Met‐Cys‐initiating N‐terminus, and showed that it positively regulates hypoxia tolerance (Gibbs *et al.*, 2018). VRN2 was coopted to this regulatory pathway in flowering plants, following duplication and N‐terminal truncation of an EMF2‐like ancestor that contained a latent internal N‐degron (Chen *et al.*, 2009; Gibbs *et al.*, 2018). Post‐translational control of VRN2 limits its accumulation in the absence of environmental stimuli that inhibit its proteolysis, including low‐O_2_ (hypoxia) and long‐term exposure to cold temperatures (Gibbs *et al.*, 2018). How this regulation of VRN2 stability contributes to its known and undescribed functions during development remains to be determined.

Hypoxia in plants occurs frequently as a result of O_2_ diffusion limitation, rapid consumption rates in tissues with high energy demands, and in response to flooding stress (Bailey‐Serres *et al.*, 2012; van Dongen & Licausi, 2015; Considine *et al.*, 2017). In plants, the transcriptional response to hypoxia is coordinated by ERFVII transcription factors, which are O_2_ and nitric oxide (NO) labile targets of the PRT6 N‐degron pathway (Gibbs *et al.*, 2011, 2014a, 2015; Licausi *et al.*, 2011). In O_2_‐replete conditions, ERFVIIs undergo a series of N‐terminal modifications, including methionine excision, cysteine oxidation and N‐terminal arginylation, which promotes their degradation by the N‐recognin E3 ligase PROTEOLYSIS6 (PRT6) (Gibbs *et al.*, 2014b, 2016; Weits *et al.*, 2014; White *et al.*, 2017). Enhanced stabilization of ERFVIIs before and during hypoxia is critical for survival in low O_2_ (Gibbs *et al.*, 2011; Licausi *et al.*, 2011; Schmidt *et al.*, 2018; Dissmeyer, 2019; Hartman *et al.*, 2019; Holdsworth *et al.*, 2019; Lin *et al.*, 2019). Furthermore, control of ERFVII stability also regulates responses to other abiotic stresses and pathogen attack (de Marchi *et al.*, 2016; Vicente *et al.*, 2017, 2019).

In addition to flooding‐induced O_2_ deprivation, endogenous hypoxic niches occur naturally in certain plant tissues, where they play a necessary and positive role in regulating development (Borisjuk & Rolletschek, 2009; Kelliher & Walbot, 2012; Meitha *et al.*, 2015, 2018; Considine *et al.*, 2017). For example, a conserved hypoxic niche in the shoot apical meristem (SAM) coordinates leaf development by constraining the accumulation of the locally expressed transcriptional regulator LITTLE ZIPPER 2 (ZPR2), a Cys‐initiating N‐degron pathway target that controls primordia formation through repressing the activity of HD‐ZIP III regulators (Weits *et al.*, 2019). A hypoxic niche is also established in lateral root primordia (LRP), triggering ERFVII stabilization to attenuate auxin signalling by inhibiting LRP developmental genes (Shukla *et al.*, 2019). Furthermore, ERFVIIs coordinate early seedling establishment during the skoto‐ to photomorphogenic transition, sensing O_2_ availability to regulate apical hook opening and limit the production of harmful Chl precursors before light is perceived (Abbas *et al.*, 2015; Zhang *et al.*, 2018). Thus, in addition to having a general role in coordinating hypoxia stress survival, ERFVIIs also have separate context‐ and tissue‐specific developmental functions.

Here we investigate how regulation of VRN2 through its O_2_‐sensitive N‐degron controls its spatiotemporal accumulation and function during development in *Arabidopsis*. Under nonstressed and ambient growth conditions, VRN2 protein is largely confined to regions of the plant that are characterized by hypoxic niches, namely the SAM and young leaf primordia (hereafter, the shoot apex) and LRPs, as well as primary and lateral root meristematic zones. Localization of VRN2 to the shoot apex modulates the photoperiod‐dependent transition to reproductive growth, independently of its role in vernalization pathways, whilst its accumulation in discrete regions of the root negatively regulates root system architecture by limiting root branching and primary root growth. Moreover, we show that ectopic stabilization of VRN2 through genetic manipulation is insufficient to trigger the vernalization response in the absence of cold exposure, as increases in other PRC2 components and cold‐specific factors are also required for appropriate silencing of *FLC* to induce flowering in response to winter. We conclude that the N‐degron of VRN2 is necessary for preventing ectopic accumulation outside of meristems and primordia, where it has specific roles in regulating growth and development. By contrast, under certain environmental conditions, including cold exposure and hypoxia, proteolysis is inhibited and VRN2 accumulates throughout the plant where, along with other context‐specific factors, it adopts a different set of developmental functions.

## Materials and Methods

### Plant growth and materials

*Arabidopsis thaliana* (L.) Heynh lines were obtained from the Arabidopsis Stock Centre (NASC), except for: Col‐0 FRI‐Sf2 (Lee *et al.*, 1994), from Dr Jie Song, Imperial College London, UK; *pVRN2::VRN2‐FLAG* and *vrn2‐1 fca‐1* (Wood *et al.*, 2006), from Dr Chris Helliwell, CSIRO, Australia; and *pFLC::FLC‐GUS* (Sheldon *et al.*, 2008), from Dr Candice Sheldon, CSIRO, Australia. The wild‐type (WT) and Cys2Ala *pVRN2::VRN2‐GUS* transgenics in Col‐0 and *prt6‐1*, as well as *vrn2‐5*, *prt6‐1*, and *prt6‐1 vrn2‐*5 lines were described previously (Gibbs *et al.*, 2018). Mutant combinations were generated by crossing, and full knockouts confirmed by PCR and reverse transcription polymerase chain reaction (RT‐PCR; primers in Supporting Information Table S1). Typically, seeds were surfaced‐sterilized in 20% Parazone, plated on half‐strength Murashige & Skoog (½MS) medium (1% agar, pH 5.7), and stratified at 4°C for a minimum of 2 d, before being transferred to long‐day (LD; 16 h : 8 h, light : dark) conditions under white fluorescent light (90–100 μmol m^−2^ s^−1^) at 22°C, and transferred to soil after 2 wk.

### Plant phenotypic analyses

For flowering time assessment*,* seedlings were grown on vertical ½MS plates for 7 d, before being transferred to soil under LD or short‐day (SD; 8 h : 16 h, light : dark, 22°C) conditions. Flowering time was determined by counting the number of rosette leaves and day number at bolting. For vernalization experiments*,* following an initial 7 d growth at 22°C, plates were transferred to SD at 5°C for the appropriate number of weeks. For nonvernalized controls, 1 wk at 5°C was correlated with 1 d growth at 22°C. Following these treatments, seedlings were harvested for protein/RNA extraction, or transferred to soil and grown under LD at 22°C until bolting, at which point rosette leaf number and day number were counted. For root assays, seedlings were grown vertically on ½MS for 10 d at 22ºC, photographed, and primary root lengths and lateral root densities (number emerged lateral roots mm^–1^ primary root) were calculated using ImageJ software (http://rsb.info.nih.gov/ij/). All phenotypic assays were performed at least three times.

### Construction of transgenic plants

The *pVRN2::VRN2‐GUS* and *pVRN2::Ala2‐VRN2‐GUS* constructs, used here to generate transgenics in the *vrn2‐ fca‐1* background, were as described previously (Gibbs *et al.*, 2018). To generate the *pER8::VIN3* construct, *VIN3* was PCR‐amplified from 2 wk vernalized Arabidopsis seedling cDNA using attB‐flanked primers, recombined into pDONOR201 using Gateway BP clonase (11789020; Invitrogen), then transferred into the destination binary vector pER8GW (Coego *et al.*, 2014) using LR clonase (11791100; Invitrogen)*.* Constructs were transformed into *Agrobacterium tumefaciens* (strain GV3101 pMP90), then transformed into relevant Arabidopsis lines using established floral dip method. At least 10 independent transgenic plants were selected for each construct; data from two independent T_3_ homozygous lines are shown.

### *In vivo* and *in vitro* protein stability analyses

Total protein was extracted from 7‐d‐old nonvernalized or vernalized seedlings as previously described (Gibbs *et al.*, 2011). To test the effect of hypoxia on *in vivo* protein stability, 7‐d‐old seedlings were exposed to 1% hypoxia for 6 or 24 h in a Heracell VIOS 160i incubator (Thermo Scientific, Waltham, MA, USA), and *ADH1* expression was used as a marker gene for hypoxia efficacy. For the β‐oestradiol induction assays, 7‐d‐old seedlings were transferred to liquid ½MS in six‐well microtitre plates supplemented with 50 µM β‐oestradiol (or equivalent volume dimethylsulphoxide control), and incubated at 22°C in the light with gentle shaking for 24 h, before harvesting in liquid nitrogen.

The VRN2‐HA *in vitro* expression construct was as previously described (Gibbs *et al.*, 2018). To generate the VIN3‐HA fusion driven by the T7 promoter, *VIN3* cDNA was PCR‐amplified from 2 wk vernalized Arabidopsis seedling cDNA and directionally cloned into a modified version of the pTNT (Invitrogen) expression vector (pTNT3xHA; Gibbs *et al.*, 2011). Cycloheximide‐chase assays were then performed using the TNT T7 Coupled Reticulocyte Lysate system (L4610; Promega) using 250 ng of each construct per 25 μl reaction as described previously (Gibbs *et al.*, 2018).

### Immunoblotting

Equal total protein amounts were resolved by sodium dodecyl sulphate‐polyacrylamide gel electrophoresis before transferring to polyvinylidene difluoride membrane via a MiniTrans‐Blot electrophoretic transfer cell (Bio‐Rad). Primary antibodies were then used to probe membranes at the following dilutions: anti‐HA (H3663; Sigma‐Aldrich), 1 : 2000; anti‐GUS (G5420; Sigma‐Aldrich), 1 : 1000. anti‐FLAG (F1804; Sigma‐Aldrich), 1 : 1000; anti‐FIE (AS12 2616; Agrisera, Vännäs, Sweden), 1 : 1000. Horseradish peroxidase‐conjugated anti‐mouse or rabbit secondary antibodies (sc‐358914 and sc‐2004; Santa Cruz, Dallas, TX, USA) were used at a titre of 1 : 10 000, before developing to film using ECL Western blotting substrate (Thermo Fisher Scientific).

### Reverse transcriptase PCR and qPCR

For semiquantitative RT‐PCR, RNA was extracted from seedlings using the RNEasy plant mini kit (74904; Qiagen). cDNA was then synthesized with Superscript II Reverse transcriptase (18064‐014; Invitrogen) using OligodT primers. PCRs were performed using gene‐ or transgene‐specific primer pairs, and *ACTIN‐2* was amplified for use as a loading control. For quantitative assessment of gene expression, RNA was extracted from seedlings (treated as described) and converted to cDNA as described earlier. Real‐time quantitative RT‐PCR was performed in triplicate using Brilliant III UF MM SYBR QPCR Low ROX master mix (600892; Agilent) on an AriaMx Real‐Time PCR system (Agilent, Santa Clara, CA, USA) according to the manufacturer’s instructions. Relative transcript abundance was determined by normalization to *ACTIN* and relative fold changes calculated. Data shown are means of three biological repeats. Error bars indicate standard deviation. For primer sequences see Table S1.

### Meristem measurements

Arabidopsis seedlings (6 d old) were stained with propidium iodide (10 μg ml^−1^) for 15 min before rinsing in water. Root meristems were visualized using a Nikon A1R Eclipse Ti inverted confocal microscope (Nikon, Tokyo, Japan). To determine meristem size, distance from the quiescent centre to the first elongated cortex cell was measured using ImageJ.

### Histochemical staining

To stain for β‐glucuronidase (GUS) enzyme activity, 7‐d‐old transgenic Arabidopsis seedlings were incubated in GUS buffer (phosphate buffer (100 mM), pH 7.0; potassium ferrycyanide (2 mM); Triton X‐100 (0.1% v/v); 1 mM X‐Gluc solution (5‐bromo‐4‐chloro‐3‐indolyl‐beta‐d‐glucuronic acid, cyclohexylammonium salt, X‐GLUC Direct)). Samples were then incubated for 4 h at 37ºC, cleared and fixed in 3 : 1 ethanol : acetic acid, then mounted on microscope slides in Hoyers solution (30 g gum Arabic, 200 g chloral hydrate, 20 g glycerol, 50 ml water) before imaging on a light microscope.

## Results

### VRN2 accumulates at the shoot apex and modulates flowering dependent on photoperiod

**Fig. 1:**
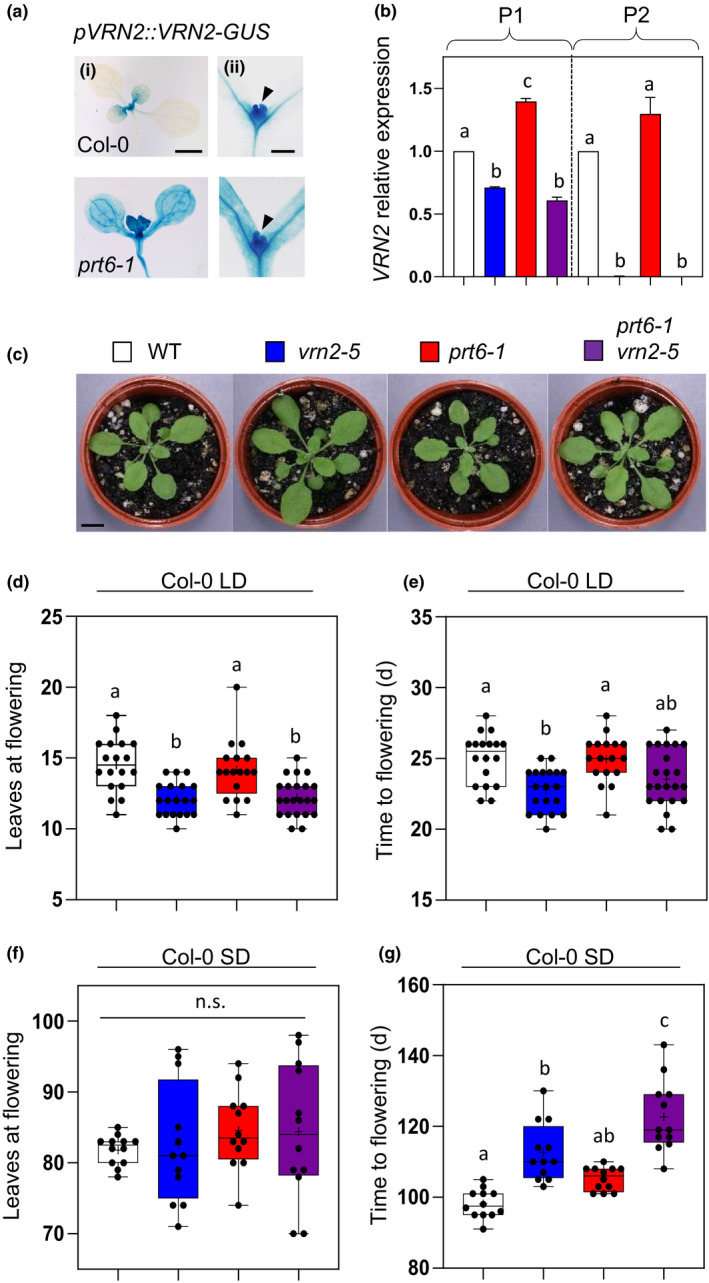
VERNALIZATION2 (VRN2) accumulates in the shoot apex and modulates flowering dependent on photoperiod. (a) Histochemical staining of Col‐0 and *prt6‐1 Arabidopsis thaliana* seedlings expressing the *pVRN2::VRN2‐GUS* translational reporter: (i) 7‐d‐old seedlings; bar, 1 mm; (ii) 4‐d‐old seedlings; bar, 200 μm. Arrowhead, shoot apical meristem. (b) Quantitative reverse transcription polymerase chain reaction (qPCR) of *VRN2* in lines indicated, using primers located upstream (P1) and downstream (P2) of the *vrn2‐5* T‐DNA insertion. Expression levels are shown relative to the wild‐type (WT) for each primer set, and ANOVA was carried out separately on P1 and P2 data. Data are average of three biological replicates. Error bars represent ±SE. For the location of P1 and P2 in *VRN2*, see Fig. 2(d). (c) Representative images of 4‐wk‐old rosettes grown under long day (LD; 16 h : 8 h, light : dark) conditions. Bar, 1 cm. (d, e) Rosette leaf number (d) and time to flowering (e) under LD conditions (*n* = 15–22 per genotype). (f, g) Rosette leaf number (f) and time to flowering (g) under short day (SD; 8 h : 16 h, light : dark) conditions (*n* = 11–13 per genotype). Box and whisker plots show maximum and minimum, 25^th^ to 75^th^ percentiles, median (horizontal line) and mean (+). Letters indicate one‐way ANOVA; Tukey’s test (*P* < 0.05–0.01).

We investigated VRN2 protein accumulation in the aerial tissues of WT Col‐0 plants using a *pVRN2::VRN2‐GUS* reporter line, which consists of full‐length VRN2 fused to GUS, driven by *c.* 2 kb of endogenous promoter (Gibbs *et al.*, 2018). Despite ubiquitous expression of *VRN2* mRNA across tissues (de Lucas *et al.*, 2016; Gibbs *et al.*, 2018), VRN2‐GUS protein was only detected in the SAM, leaf primordia, young expanding leaves, and parts of the vasculature (Fig. 1a), which correlates with a previous study that showed enrichment of VRN2‐FLAG at the shoot tip (Wood *et al.*, 2006). This localization is remarkably similar to that observed for a *pHRPEx5:GFP‐GUS* hypoxia reporter construct expressed in Col‐0 seedlings of a similar age (Weits *et al.*, 2019). By contrast, *pVRN2::VRN2‐GUS* in the *prt6‐1* mutant accumulated throughout all tissues of the seedling, resembling *pHRPEx5:GFP‐GUS* expression in lines grown under 5% O_2_ (Weits *et al.*, 2019). Quantitative PCR analysis confirmed that *VRN2* expression was not significantly increased in *prt6‐1* relative to WT (Fig. 1b). We therefore conclude that the N‐degron pathway restricts VRN2 to the hypoxic shoot apex in the aerial tissues of Arabidopsis seedlings.

**Fig. 2:**
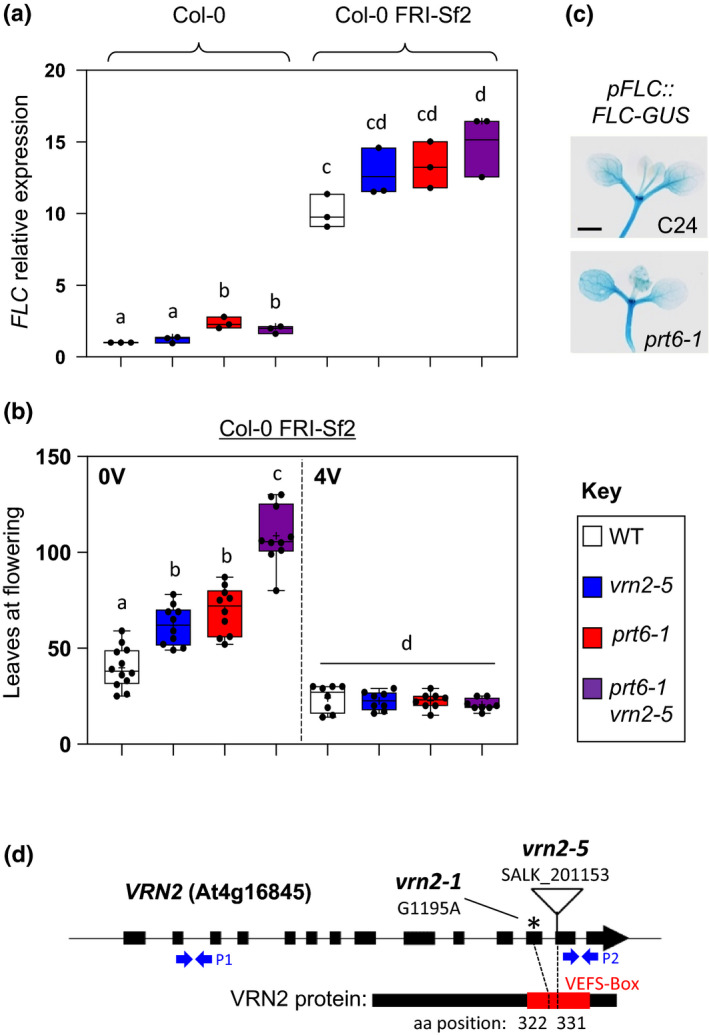
VERNALIZATION2 (VRN2) function in vernalization‐dependent lines. (a) Quantitative reverse transcription polymerase chain reaction (qPCR) of *FLC* in Col‐0 and Col‐0 FRI‐Sf2 wild‐type (WT) and mutant *Arabidopsis thaliana* backgrounds. Expression levels are shown relative to Col‐0 WT. Data are means of three biological replicates. (b) Rosette leaf number at flowering in Col‐0 FRI‐Sf2 WT and mutant lines under nonvernalizing (0V) or 4‐wk‐vernalizing (4V) conditions (*n* = 8–12 per genotype). (c) Histochemical staining of the *pFLC::FLC‐GUS* translational reporter in C24 (WT) and *prt6‐1*. Bar, 1 mm. (d) Schematic diagram of the *VRN2* locus and the derived protein, showing the functionally important C‐terminal VEFS‐box domain. The location of *vrn2‐1* and *vrn2‐5* mutations is indicated along with the predicted amino acid (aa) positions disrupted in VRN2. Blue arrows show position of primer pairs used for qPCR in Fig.1(b). The asterisk highlights the position of *vrn2‐1* point mutation. Box and whisker plots in (a) and (b) show maximum and minimum, 25^th^ to 75^th^ percentiles, median (horizontal line) and mean (+). Letters indicate one‐way ANOVA; Tukey’s test (*P* < 0.05–0.01).

To investigate how control of VRN2 stability by the N‐degron pathway regulates its functions in the shoot, we took advantage of a range of genetic mutants in the Col‐0 background: *vrn2‐5* (which lacks full‐length VRN2), *prt6‐1* (which ectopically accumulates VRN2, as well as other N‐degron targets including ERFVIIs and presumably ZPR2), and *prt6‐1 vrn2‐5* (which accumulates all PRT6 N‐degron substrates except for VRN2). We designed *VRN2*‐specific primers upstream and downstream of the *vrn2‐5* insertion site, and confirmed that the T‐DNA completely abolished expression of full‐length *VRN2* mRNA, although a C‐terminally truncated mRNA could still be detected at reduced levels compared with the WT (Figs 1b, 2d). When these lines were grown under LD conditions at 22ºC, no obvious effects on phyllotaxis or morphology were observed, except for some small variations in rosette size (Fig. 1c). However, both *vrn2‐5* and *prt6‐1 vrn2‐5* flowered earlier than Col‐0 and *prt6‐1* when leaf number at bolting and time to flowering were assessed (Fig. 1d,e). This indicates a repressive function for VRN2 in flowering, similar to reported roles for VRN2‐like proteins in *Medicago* (Jaudal *et al.*, 2016). Whilst loss of VRN2 reduced time to flowering, *prt6‐1* did not exhibit delayed flowering relative to WT, suggesting that ectopic accumulation of VRN2 is insufficient to enhance its effects on this process.

Col‐0 is a facultative LD plant, with short photoperiods having a strong repressive effect on reproductive transition. Therefore, we also assessed flowering phenotypes under SD conditions at 22ºC. Here, all three mutants had reduced developmental synchronicity relative to WT when leaf number at bolting was scored (i.e. broader variation in leaf numbers) (Fig. 1f). When chronological timing of flowering was determined, both *vrn2‐5* and *prt6‐1* flowered significantly later than Col‐0, with the double mutant displaying an even stronger delay (Fig. 1g). This indicates additive positive roles in flowering for VRN2 and PRT6 under SD conditions, although a more general pleiotropic effect on quiescence cannot be ruled out. For *vrn2‐5* this is opposite to what was observed under LD conditions, revealing distinct photoperiod‐dependent roles for VRN2 in the shoot (Fig. 1d,e). The ERFVIIs have previously been shown to positively regulate flowering (Vicente *et al.*, 2017), which probably explains the *prt6‐1* phenotype observed here. Our data reveal that VRN2 has multiple roles in regulating flowering that are separate from its role in vernalization, dependent on photoperiod, and not enhanced when its levels are ectopically increased through genetic manipulation.

### VRN2 function in vernalization‐dependent lines

VRN2 was initially identified as a positive regulator of the vernalization response in late‐flowering Arabidopsis mutants (Chandler *et al.*, 1996). Col‐0 is an early‐flowering ecotype that does not require vernalization as a result of an inactive allele of *FRIGIDA* (Johanson *et al.*, 2000), a positive regulator of *FLC* expression. To investigate how post‐translational control of VRN2 stability contributes to its functions in the vernalization response, we crossed the double *prt6‐1 vrn2‐5* mutant to the late‐flowering Col‐0 FRI‐Sf2 introgression line (hereafter FRI‐Sf2), which is almost identical to Col‐0 except that it contains a dominant *FRIGIDA* allele derived from the Sf2 ecotype (Johanson *et al.*, 2000). Single and double mutants in the FRI‐Sf2 background were then identified in the F_2_ generation (Fig. S1). We confirmed increased levels of *FLC* expression in FRI‐Sf2 relative to Col‐0 using qPCR, and observed a concomitant delay in flowering under LD conditions (*c.* 40 leaves vs 14 in Col‐0) (Fig. 2a,b). Both *vrn2‐5* FRI‐Sf2 and *prt6‐1* FRI‐Sf2 had slightly higher levels of *FLC* expression relative to WT FRI‐Sf2, with the double mutant having the greatest increase (Fig. 2a). This corroborates previous work showing elevated *FLC* expression in late‐flowering mutants carrying the *vrn2‐1* mutation (Sheldon *et al.*, 2000), and indicated that single *vrn2‐5* and double *prt6‐1 vrn2‐5* mutants in the FRI‐Sf2 background would flower later than the WT, which we observed when we grew them under LD conditions (Fig. 2b). Remarkably, the *prt6‐1 vrn2‐5* FRI‐Sf2 line flowered extremely late relative to FRI‐Sf2 (> 100 leaves vs *c.* 40), suggesting a similar additive role for VRN2 and PRT6 in promoting flowering to that observed in the Col‐0 ecotype under SD conditions (Fig. 1d,e). Increased *FLC* expression and delayed flowering in *prt6‐1* FRI‐Sf2 indicate that ectopic accumulation of VRN2 in the absence of cold exposure is insufficient to repress *FLC* and abolish a requirement for vernalization. Further supporting this, activity of *pFLC::FLC‐GUS* was not reduced in *prt6‐1* relative to a vernalization‐dependent C24 parental line (Fig. 2c).

We exposed all FRI‐Sf2 lines to 4 wk vernalization (4V) treatment at the seedling stage (8 h : 16 h, light : dark, 5ºC), before returning to LD conditions at 22ºC and assessing leaf number at bolting. Remarkably, all four lines flowered significantly earlier in response to this treatment (Fig. 2b). This was particularly striking for the *prt6‐1 vrn2‐5* FRI‐Sf2 mutant (from > 100 leaves to < 30). Thus, the repressive effects induced by loss of VRN2 and PRT6 activity can be overridden by exposure to low temperatures. This was unexpected for *vrn2‐5*, as VRN2 is required for vernalization, and the previously isolated *vrn2‐1* mutant allele is insensitive to cold exposure (Sheldon *et al.*, 2000; Gendall *et al.*, 2001). The *vrn2‐1* mutant was isolated from an EMS screen for plants that do not respond to vernalization (Chandler *et al.*, 1996), and contains a codon substitution that leads to premature truncation of VRN2 at amino acid 322 (Gendall *et al.*, 2001). By contrast, *vrn2‐5* has a T‐DNA insertion that is predicted to disrupt the gene downstream of the *vrn2‐1* mutation site, at a residue encoding amino acid 331, and qPCR analysis confirmed that a truncated *VRN2* mRNA is expressed (Fig. 1b). Both mutations occur in the VEFS‐box domain of VRN2, a critical region of the protein that is conserved in Su(z)12 homologues and required for facilitating binding and catalytic function of PRC2 (Fig. 2d) (Cao & Zhang, 2004; Ketel *et al.*, 2005). Given that *vrn2‐5* has clear developmental defects related to flowering and root growth (see later), our data suggest that the *vrn2‐5* allele disrupts some VRN2 functions, but, in contrast to *vrn2‐1*, does not abolish vernalization capacity.

### Ectopic stabilization of VRN2 does not abolish the requirement for vernalization

**Fig. 3:**
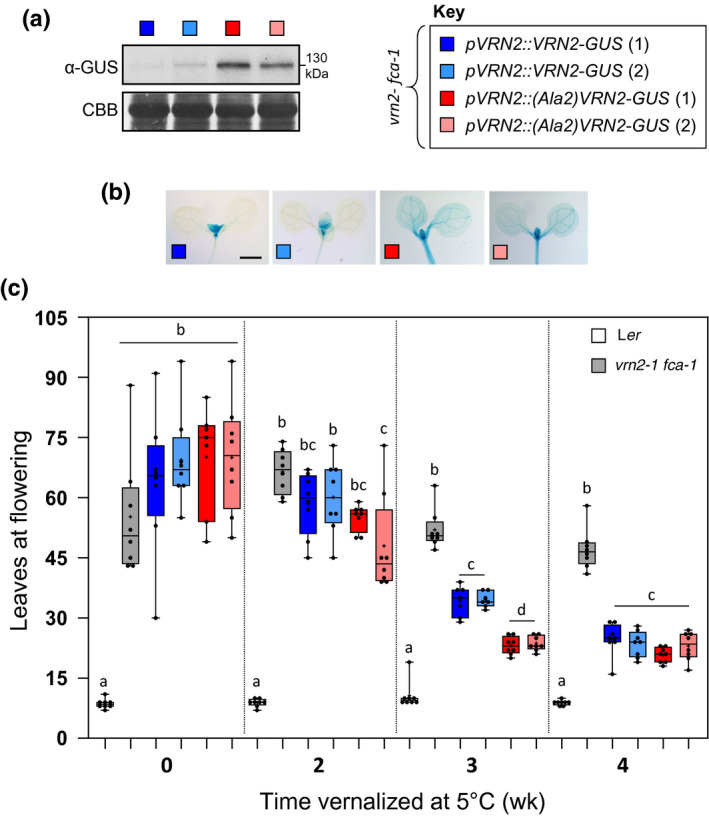
Ectopic stabilization of VERNALIZATION2 (VRN2) does not abolish the requirement for vernalization. (a, b) Anti‐β‐glucuronidase (anti‐GUS) Western blot (a) and histochemical staining (b) of 7‐d‐old *vrn2‐1 fca‐1 Arabidopsis thaliana* seedlings expressing either wild‐type (WT) or mutant (Ala2) *pVRN2::VRN2‐GUS*. CBB, Coomassie brilliant blue. Bar, 1 mm. (c) Rosette leaf number at flowering for Landsberg *erecta* (L*er*), *vrn2‐1 fca‐1*, and *vrn2‐ fca‐1* expressing WT or mutant (Ala2) *pVRN2::VRN2‐GUS* under nonvernalization conditions (0 wk) or following 2–4 wk vernalization (*n* = 8–10 per genotype per condition). Box and whisker plots in (c) shows maximum and minimum, 25^th^ to 75^th^ percentiles, median (horizontal line) and mean (+). Letters indicate one‐way ANOVA; Tukey’s test (*P* < 0.05–0.01).

We next investigated how regulation of VRN2 stability influences vernalization using the *vrn2‐1* allele, which is in the late‐flowering *fca‐1* mutant in Landsberg *erecta* (L*er*) (Chandler *et al.*, 1996). FCA is a component of the autonomous flowering pathway that regulates RNA‐mediated chromatin silencing (Baurle *et al.*, 2007); the *fca‐1* mutant has high levels of *FLC* expression, leading to a late‐flowering phenotype that can be overcome by vernalization. Whilst *vrn2‐1 fca‐1* is in the L*er* ecotype, *prt6‐1* is in Col‐0. Owing to a lack of *prt6* mutants in L*er*, and to avoid mixing ecotypes, we instead ectopically stabilized VRN2 by introducing WT (Cys2) or mutant (Cys2Ala) variants of *pVRN2::VRN2‐GUS* into *vrn2‐1 fca‐1*. Western blotting and histochemical staining confirmed that the Cys2Ala mutation in VRN2 is sufficient to enhance its abundance and expand its domain of accumulation throughout the seedling, similar to *prt6‐1,* whilst WT VRN2 showed characteristic localization to the hypoxic shoot apex (Fig. 3a,b; two independent lines for each transgene).

We investigated flowering phenotypes in these transgenic lines relative to the untransformed *vrn2‐1 fca‐1* parent line. WT *pVRN2::VRN2‐GUS* and mutant *pVRN2::Ala2‐VRN2‐GUS* plants all flowered late under LD conditions, similar to the *vrn2‐1 fca‐1*, with all lines showing low synchronicity in leaf number at bolting (Fig. 3c). We exposed these lines to increasing lengths of time at 5°C (2, 3 and 4 wk). As expected, the *vrn2‐1 fca‐1* mutant was insensitive to vernalization treatment. However, all four transgenics flowered earlier in a dose‐dependent manner, signifying that the WT and mutant *pVRN2::VRN2‐GUS* constructs can functionally compensate for the *vrn2‐1* mutation. Extended exposure to cold also led to greater synchronicity of flowering for all lines tested. The mutant *pVRN2::Ala2‐VRN2‐GUS* plants had a slightly enhanced response to shorter cold exposure times, which was most pronounced following 3 wk of vernalization. However, by 4 wk all transgenic lines flowered at a similar time. We therefore conclude that ectopic stabilization of VRN2 by mutating its N‐degron is insufficient to significantly enhance its function during vernalization relative to WT.

### VRN2 stability in relation to other PRC2 components and the VIN3 co‐factor

Our data reveal that VRN2 accumulation in *prt6‐1* or through N‐terminal mutagenesis does not lead to increased VRN2 function with regard to photoperiod‐ or vernalization‐associated flowering. The former could be explained by the fact that VRN2 is already stabilized in regions of the plant (i.e. hypoxic niches) where this function is established, with enhanced abundance outside these domains having no further influence. The latter is probably a result of a lack of other cold‐specific factors that are required for efficient silencing of *FLC* (Costa & Dean, 2019). Nonetheless, these findings prompted us to investigate the relationship between VRN2 stability and the abundance of other core PRC2 components and accessory proteins during cold exposure, hypoxia, and in the *prt6‐1* mutant.

**Fig. 4:**
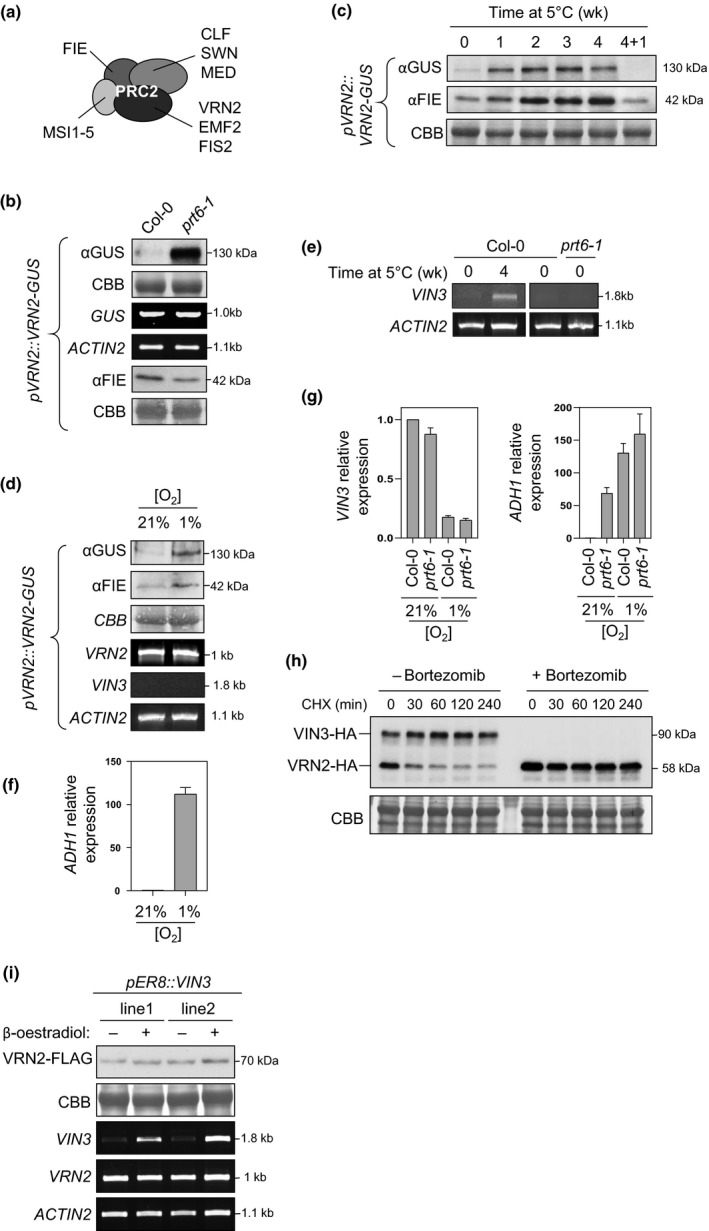
VERNALIZATION2 (VRN2) stability in relation to other PRC2 subunits and the VIN3 cofactor. (a) Diagram showing *Arabidopsis thaliana* proteins making up the four core subunits of PRC2. (b) Steady‐state protein and mRNA levels of VRN2‐GUS and FERTILIZATION INDEPENDENT ENDOSPERM (FIE) in Col‐0 and *prt6‐1* expressing *pVRN2::VRN2‐GUS*. (c) Steady‐state protein abundance of VRN2‐GUS and FIE in vernalized (0–4 wk) Col‐0 seedlings expressing *pVRN2:VRN2‐GUS*. 4 + 1 refers to 1 wk recovery at 22°C following 4 wk cold exposure. (d) Steady‐state protein and mRNA levels of VRN2‐GUS, FIE, and *VIN3* in 7‐d‐old *pVRN2::VRN2‐GUS* seedlings under normoxia (21% O_2_) or following 6 h hypoxia (1% O_2_) treatment. (e) Reverse transcription polymerase chain reaction (RT‐PCR) of *VIN3* in Col‐0 or *prt6‐1* ± 4 wk vernalization treatment. (f) Quantitative RT‐PCR (qPCR) of *ADH1* for samples in (d), confirming efficacy of hypoxia treatment. Data are means of three biological replicates. Error bars represent ± SE. (g) qPCR of *VIN3* and *ADH1* in Col‐0 and *prt6‐1* under normoxia (21% O_2_) or following 24 h hypoxia (1% O_2_) treatment. Data are means of three biological replicates. Error bars represent ± SE. (h) *In vitro* cycloheximide (CHX) chase time course of VRN2‐HA coexpressed with VIN3‐HA, or coincubated with the proteasome inhibitor bortezomib in the absence of VIN3‐HA. (i) Steady‐state levels of VRN2‐FLAG and FIE in *pVRN2::VRN2‐FLAG* lines expressing an β‐oestradiol‐inducible VIN3 construct (*pER8::VIN3*). Two independent lines treated with or without β‐oestradiol are shown, and RT‐PCR shows relative levels of *VIN3*, *VRN2* and *ACTIN2* expression.

The Arabidopsis PRC2 complex consists of four core subunits; three of these are encoded by multiple family members, but one of these (FIE) is not (Fig. 4a). We therefore used FIE protein as a proxy for relative PRC2 abundance in relation to VRN2 accumulation. Western blot analysis showed that whilst VRN2 accumulated to high levels in *prt6‐1* relative to WT, the amount of total FIE protein was unaltered (Fig. 4b). During cold exposure, however, FIE abundance increased in a dose‐dependent manner, and depleted again upon return to warm temperatures, similar to VRN2 (Fig. 4c). It was previously also shown that CLF and SWN methyltransferases accumulate during cold exposure (Wood *et al.*, 2006). We also saw similar increases in both VRN2‐GUS and FIE abundance in response to hypoxia (Fig. 4d). This suggests that genetic enhancement of VRN2 levels (i.e. in *prt6‐1*) might be insufficient to stimulate enhanced VRN2‐PRC2 activity because of a lack of a concomitant increase of other complex subunits or interaction partners, which only occurs when VRN2 accumulates in environmental contexts (i.e. cold exposure or hypoxia).

We also examined the dynamics of the VRN2‐PRC2 cofactor VIN3. VIN3 is a key player in the vernalization response (Sung & Amasino, 2004), which is transcriptionally induced by cold temperatures and binds specifically to VRN2‐PRC2 along with VRN5 to potentiate methylation of the *FLC* nucleation site (Greb *et al.*, 2007; Costa & Dean, 2019). Interestingly, *VIN3* was also previously shown to be upregulated by hypoxia, where it contributes to hypoxia resilience, similar to VRN2 (Bond *et al.*, 2009; Gibbs *et al.*, 2018). We confirmed cold‐responsive induction of *VIN3* expression (Fig. 4e). However, we found that 6 h exposure to 1% O_2_ was not sufficient to induce *VIN3*, despite enhancing VRN2‐GUS stability and increasing *ADH1* expression > 100‐fold, indicating different timescales for regulation in response to O_2_ deprivation (Fig. 4d,f). To test if longer hypoxic treatments trigger *VIN3* induction, we exposed both WT and *prt6‐1* seedlings to 1% O_2_ for 24 h. However, we saw a reduction rather than an increase in *VIN3* expression, despite *ADH1* mRNA levels confirming the efficacy of the hypoxia treatment (Fig. 4g). Furthermore, *VIN3* mRNA levels were not elevated in *prt6‐1* relative to WT (Fig. 4e,g), and *VIN3* does not appear in published hypoxia microarray datasets (Gibbs *et al.*, 2011), indicating that transcriptional control of *VIN3* is not linked to the canonical mechanism for hypoxia‐responsive gene induction through ERFVIIs. As such, a mechanistic connection between VRN2 and *VIN3* under hypoxia is still unclear.

It had previously been proposed that cold‐triggered induction of *VIN3* (Fig. 4e) might enhance the abundance of PRC2 subunits during vernalization through binding and stabilizing the complex (Wood *et al.*, 2006). We tested the possibility that VIN3 promotes VRN2 stability (e.g. through steric shielding of the VRN2 N‐degron) by coexpressing VIN3‐HA and VRN2‐HA in a cell free rabbit reticulocyte system that contains a functional Arg N‐degron pathway (Gibbs *et al.*, 2011), and monitoring protein abundance over time following treatment with the translational inhibitor cycloheximide (CHX) (Fig. 4h). Here, VRN2‐HA was unstable even in the presence of VIN3‐HA. By contrast, when VRN2‐HA was co‐incubated with the proteasome inhibitor bortezomib without VIN3‐HA, its turnover was inhibited. Thus, VIN3 alone is not sufficient to stabilize VRN2. We also introduced β‐oestradiol‐inducible *VIN3* constructs into the previously described *pVRN2::VRN2‐FLAG* Arabidopsis line, to test if ectopic induction of *VIN3* *in planta* affects VRN2 abundance. However, despite clear induction of *VIN3* expression in the presence of β‐oestradiol, no obvious increase in VRN2‐FLAG stability was observed (Fig. 4i). Together, these data suggest that whilst VIN3 is required for vernalization through its association with VRN2‐PRC2, it does not promote increased stability of VRN2.

### The PRT6 N‐degron pathway confines VRN2 to discrete root tissues and negatively regulates root growth

**Fig. 5:**
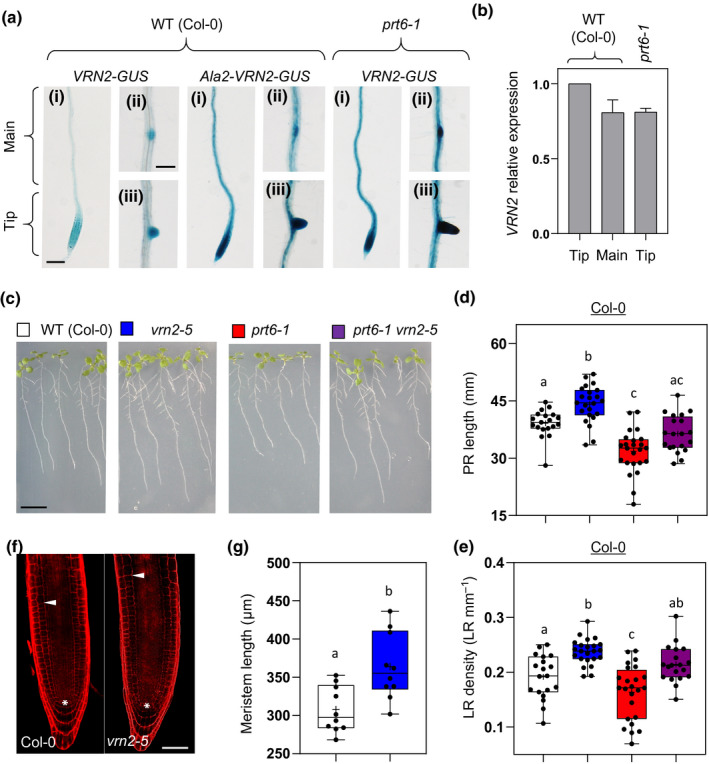
The PRT6 N‐degron pathway confines VERNALIZATION2 (VRN2) to discrete root tissues and negatively regulates root growth. (a) Histochemical β‐glucuronidase (GUS) staining of 7‐d‐old roots in Col‐0 and *prt6‐1 Arabidopsis thaliana* seedlings expressing wild‐type (WT) or mutant (Ala2) *pVRN2::VRN2‐GUS*: (i) primary root (PR) tip; bar, 1 mm; (ii) lateral root primordium (LRP); bar, 500 μm; (iii) emerged LR. (b) Quantitative reverse transcription polymerase chain reaction (qPCR) of VRN2 in different regions of WT or *prt6‐1* primary roots. The tip and main regions are shown in (a). Data are means of three biological replicates. Error bars represent ± SE. (c) Representative images of 10‐d‐old WT and mutant seedling roots in the Col‐0 background. Bar, 1 cm. (d, e) Quantified PR lengths (d) and emerged LR densities (e) of 10‐d‐old Col‐0 WT and mutant lines (*n* = 13–24). (f) Representative images of 6‐d‐old Col‐0 and *vrn2‐*5 primary root meristems stained with propidium iodide. Asterisk represents the quiescent centre; arrowhead indicates the end of the meristem. Bar, 100 μm. (g) Quantification of meristem size in Col‐0 and *vrn2‐5*. Data calculated from confocal root images (*n* = 10). Box and whisker plots show maximum and minimum, 25^th^ to 75^th^ percentiles, median and mean (+). Letters indicate one‐way ANOVA; Tukey’s test (*P* < 0.05–0.01).

In addition to assessing the spatiotemporal pattern of VRN2 localization in aerial tissues, we investigated VRN2 abundance in the root system of seedlings. WT VRN2‐GUS was detected in the primary root (PR) meristem zone, in LRPs and emerged lateral roots (LRs), and parts of the vasculature (Fig. 5a). By contrast, mutant Ala2‐VRN2‐GUS, and WT VRN2‐GUS in *prt6‐1*, had expanded domains of accumulation, localizing throughout the root (Fig. 5a). Quantitative RT‐PCR analysis confirmed that *VRN2* expression levels are not enhanced in WT root tips relative to the main root, or in *prt6‐1* root tips compared with the WT (Fig. 5b). Thus, similar to aerial tissues, the N‐degron pathway post‐translationally restricts VRN2 protein to discrete regions of roots.

Mid‐to‐late stage LRPs have recently been shown to be hypoxic (Shukla *et al.*, 2019). Interestingly, in contrast to the SAM, the root meristem of young, establishing seedlings may not be hypoxic, as anaerobic gene expression is not enriched in this region and the ERFVII RAP2.12 does not accumulate there (Hartman *et al.*, 2019; Weits *et al.*, 2019). However, in the PR tip of older seedlings, ERFVIIs are stable (Holdsworth *et al.*, 2019). Thus, as VRN2 mRNAs are not enriched in this region relative to other parts of the root (Fig. 5b), the accumulation of VRN2 protein may be a result of other factors blocking VRN2 proteolysis, differential sensitivities to O_2_ availability in this tissue, or age‐dependent variability in N‐degron pathway activity (Giuntoli *et al.*, 2017).

Given the localization of VRN2 to LRPs and the PR meristem, we investigated root architecture in *vrn2‐5*, *prt6‐1* and *prt6‐1 vrn2‐5* relative to Col‐0. When grown on vertical agar plates, seedlings of the *vrn2‐5* mutant had significantly longer PRs than those of the WT, whilst *prt6‐1* mutant roots were shorter (Fig. 5c,d). The *prt6‐1 vrn2‐*5 mutant had PRs of a similar length to the WT, indicating that stable VRN2 contributes to the reduced root length phenotype of *prt6‐1*. We also observed enhanced meristem size in *vrn2‐5* relative to the WT, which correlates with the increased PR lengths observed in this line (Fig. 5f,g). A similar pattern across the mutants was observed when emerged LR densities were scored: *vrn2‐5* had increased LR density, *prt6‐1* reduced density, and the double mutant had an intermediate phenotype (Fig. 5e). *prt6‐1* was recently shown to have reduced LR density as a result of an accumulation of ERFVIIs, which repress LR production (Shukla *et al.*, 2019). Our data suggest that repression of LRs in *prt6‐1* is controlled by stable VRN2 as well as ERFVIIs.

**Fig. 6:**
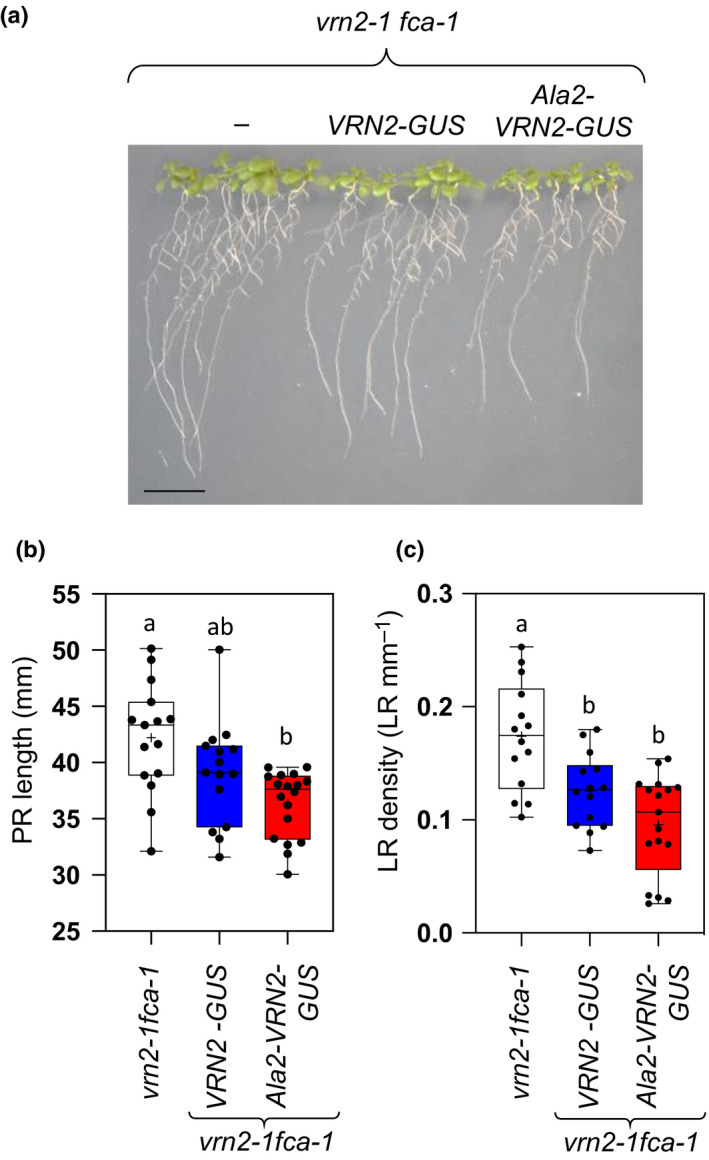
Ectopic stabilization of VERNALIZATION2 (VRN2) enhances its negative effects on root system architecture. (a) Representative images of 10‐d‐old *vrn2‐1 fca‐1*, *pVRN2::VRN2‐GUS* and *pVRN2::Ala‐VRN2‐GUS Arabidopsis thaliana* lines. Bar, 1 cm. (b, c) Quantified primary root (PR) lengths (b) and emerged lateral root (LR) densities (c) for *vrn2‐1 fca‐1*, and *vrn2‐1 fca‐1* transformed with *pVRN2::VRN2‐GUS* or *pVRN2::Ala‐VRN2‐GUS* lines (*n* = 15–18). Box and whisker plots show maximum and minimum, 25^th^ to 75^th^ percentiles, median and mean (+). Letters indicate one‐way ANOVA; Tukey’s test (*P* < 0.05–0.01).

To further investigate the role of VRN2 in regulating root system architecture, we also examined root growth in the *vrn2‐1 fca‐1* mutant, as well as *vrn2‐1 fca‐1* complemented with WT *pVRN2::VRN2‐GUS* or mutant stable *pVRN2::Ala2‐VRN2‐GUS* (Fig. 3a,b). Here, both transgenes led to a reduction in PR length and emerged LR density relative to *vrn2‐1 fca‐1*, but this was most pronounced in the *pVRN2::Ala2‐VRN2‐GUS* line (Fig. 6a–c). This therefore corroborates our findings in Col‐0, identifying VRN2 as a negative regulator of root growth, and indicates that, in contrast to flowering, ectopic stabilization of VRN2 is sufficient to enhance its function in roots.

## Discussion

Here we investigated how control of VRN2 by the PCO‐dependent branch of the PRT6 N‐degron pathway relates to its localization and functions in plant development. Our data indicate that post‐translational control of VRN2 plays a key role in restricting its accumulation to specific regions of the shoot and root that are hypoxic, where it contributes to the regulation of flowering time and repression of root growth. These roles are separate from its function in vernalization, which is potentiated in response to cold temperatures that inhibit proteolysis to enhance VRN2 abundance throughout the plant. Thus, our findings suggest that the N‐degron of VRN2 has a distinct role in limiting VRN2 abundance to discrete tissues, whilst also permitting accumulation in response to environmental inputs where it carries out a different set of context‐specific functions.

In the early‐flowering Col‐0 ecotype, VRN2 has opposing roles in modulating flowering, dependent on photoperiod: under LD conditions VRN2 is repressive, whilst under SD conditions it has a positive function (Fig. 1). In late‐flowering FRI‐Sf2, the positive function of VRN2 also manifests under LD conditions (Fig. 2). We found that ectopic stabilization of VRN2 did not enhance the photoperiod‐dependent functions of VRN2 (Fig. 3). This is probably because such functions are linked to VRN2 activity in the shoot apex, where it is already stable as a result of maintenance of a hypoxic niche in this region. How VRN2 differentially influences flowering remains to be determined. Photo‐period dependent flowering is regulated by a complex network of floral regulators, which includes components of the circadian clock and light receptors, which converge on the zinc finger transcription factor *CONSTANS* that, in turn, modulates levels of the Florigen gene *FLOWERING LOCUS T* (Song *et al.*, 2015). VRN2 may contribute to the epigenetic regulation of any or several components in this pathway, or alternatively it could have pleiotropic effects on this developmental process related to growth and quiescence. Future analysis of genome‐wide methylation targets of VRN2‐PRC2 may shed light on this.

VRN2 also had a restricted pattern of accumulation in root tissues, where the PCO branch of the PRT6 N‐degron pathway limits its abundance to the root meristem zone and LRPs to repress root development (Figs 5, 6). Plants with mutations in VRN2 (*vrn2‐5* and *vrn2‐1*) had increased PR lengths and emerged LR densities, whilst the *prt6‐1* mutant and plants expressing stable Ala2‐VRN2‐GUS had opposite root phenotypes (i.e. shorter PRs and a reduced LR density). This effect was partially reverted in the *prt6‐1 vrn2‐5* double mutant, indicating that ectopic accumulation of VRN2 in roots does lead to enhanced function, in contrast to the situation in aerial tissues. It was previously reported that different PRC2 subunits have distinct and opposing roles in the control of root development: mutations in SWN and MSI1 cause smaller PRs with reduced meristem size, whilst a CLF mutant (*clf29*) had longer roots and significantly increased numbers of cells in the meristem (de Lucas *et al.*, 2016). We observed enhanced meristem size in *vrn2‐5* relative to WT, which is similar to the previous observation in *clf29*. Thus, it is possible that the repressive role of VRN2 in root system architecture is linked to the CLF methyltransferase.

Hypoxic niches have recently been identified in pre‐emerged LRPs, which probably explains why VRN2 accumulates in these regions (Shukla *et al.*, 2019). However, in contrast to the shoot meristem, root meristems are yet to be defined as hypoxic when assayed in normoxia (Weits *et al.*, 2019), and so it is possible that VRN2 accumulation in the root tip is linked to alternative mechanisms inhibiting its proteolysis (e.g. perhaps steric shielding of the N‐degron by a tissue‐specific binding partner). Alternatively, root meristems may show different sensitivity to O_2_, as root tip growth occurs in the soil, which is likely to be a hypoxic environment (Abbas *et al.*, 2015). Hypoxic niches in LRPs were recently shown to enhance ERFVIIs, which inhibit LR development through repressing the expression of the auxin‐associated genes *LBD16/18*, *IAA29* and *PUCHI* (Shukla *et al.*, 2019). It will be important to determine if any of these same genes are also repressed at the epigenetic level through the action of locally stabilized VRN2, or whether separate targets are involved.

VRN2 is well characterized as a positive regulator of the vernalization response, accumulating during cold exposure to facilitate methylation and silencing of *FLC*. However, ectopic accumulation of VRN2 (in either *prt6‐1* or through N‐terminal mutagenesis) did not repress *FLC* or abolish the requirement for vernalization (Figs 2, 3). This was perhaps not unexpected, as other cold‐specific proteins and regulatory lncRNAs are required for the epigenetic repression of *FLC* (Costa & Dean, 2019). However, this led us to investigate in further detail the relationship between VRN2 abundance and the presence of other PRC2 subunits that are necessary for VRN2 to carry out PRC2‐associated functions. When VRN2 is ectopically enhanced, levels of the core PRC2 component FIE do not change (Fig. 4), which indicates an overaccumulation of ‘free’ VRN2 protein. By contrast, cold‐ and hypoxia‐triggered increases in VRN2 abundance were accompanied by higher levels of FIE. Thus, when VRN2 is stabilized outside of meristems in response to environmental signals, there is the capacity for a similar overall increase in VRN2‐PRC2 that cannot take place when VRN2 accumulates out of context. The PHD protein VIN3 was previously shown to be transcriptionally induced by both cold and hypoxia, two environmental conditions that also inhibit VRN2 proteolysis. Whilst we also observed cold induction of *VIN3* transcripts, we did not see an increase in *VIN3* expression in response to hypoxia treatment (Sung & Amasino, 2004; Bond *et al.*, 2009; Gibbs *et al.*, 2018). However, it should be noted that here we used 1% O_2_, whereas Bond *et al.* (2009) used 0.1%, suggesting that perhaps *VIN3* induction requires extremely low O_2_ availability, or even anoxia. *VIN3* expression was not increased in *prt6‐1* relative to Col‐0, further highlighting that VRN2‐PRC2 binding partners required for vernalization are not available when VRN2 artificially accumulates. It was previously postulated that VIN3 might promote VRN2‐PRC2 increases during long‐term cold exposure, through binding and enhancing stability of the complex. We explored this possibility by investigating the effect of VIN3 on VRN2 stability *in vitro* and *in planta*. VIN3 did not stabilize VRN2, which suggests alternative mechanisms promoting observed increases in PRC2 components in response to cold temperatures (Fig. 4; Wood *et al.*, 2006). Collectively, our data indicate that functions for VRN2 outside of meristems are only activated when it accumulates in appropriate environmental contexts as a result of a requirement for other specific binding factors.

The *vrn2‐5* mutant used in this study displayed several phenotypes related to both flowering and root development. This mutant was also previously shown to influence hypoxia tolerance and maternal effects on seed dormancy (Auge *et al.*, 2017; Gibbs *et al.*, 2018). However, in contrast to the *vrn2‐1*, *vrn2‐5* was still able to fully respond to vernalization treatment, indicating that *vrn2‐5* disrupts some but not all VRN2 functions. The VEFS domain in Su(z)12 is required for binding to PRC2 and stimulating methyltransferase activity (Cao & Zhang, 2004). Full deletion of the VEFS domain abolishes the capacity for Su(z)12 to associate with catalytic Ez subunit in Drosophila. By contrast, a series of point mutations at different positions in the VEFS box of Su(z)12 affected PRC2 activity to different degrees (Ketel *et al.*, 2005). A D593A mutation in the latter half of the VEFS‐box had no effect on PRC2 assembly or enzymatic function, whilst a D550A mutation earlier in the sequence had a modest effect on methyltransferase activity (Ketel *et al.*, 2005). However, an E546A mutation just four residues upstream of D550 almost completely abolished PRC2 function. Thus it is plausible that the different positions of mutation in *vrn2‐1* and *vrn2‐5* could differentially affect VRN2 activity, perhaps through modulating binding stoichiometries, as has been observed previously for other Su(12z) mutations (Birve *et al.*, 2001). It will now be important to develop further knockouts of VRN2 in different ecotypes (e.g. through the use of CRISPR) to help dissect its different functions in development and environmental response.

In conclusion, we show that VRN2 has multiple functions in plant development that are linked to the control of its abundance through the PCO branch of the PRT6 N‐degron pathway. Cooption of Su(z)12 to this proteolytic system allows plants to control spatial abundance and function of VRN2 by limiting it to endogenous hypoxic niches, whilst also coupling its accumulation to the perception of specific environmental cues where it adopts a separate set of functions. In this way, regulation of VRN2 by the PRT6 N‐degron pathway is similar to that for ERFVIIs, which also have dual functionality in tissue‐specific coordination of development, and broader environment‐triggered regulation of stress responses.

## Author contributions

DJG, A‐ML and MJH designed and conceived the research. DJG, A‐ML, HMT, MB, CS, RDE, GK, OA and MJH conducted experiments. DJG, A‐ML and MJH analysed data. DJG wrote the manuscript with input from all authors.

## Supporting information


**Fig. S1** Genotyping PCR confirming homozygosity of mutants in the Col‐0 FRI‐Sf2 background.
**Table S1** Primer sequences used in this study.
Please note: Wiley Blackwell are not responsible for the content or functionality of any Supporting Information supplied by the authors. Any queries (other than missing material) should be directed to the *New Phytologist* Central Office.
