# Supplementary material for: The PRT6 N‐degron pathway restricts VERNALIZATION 2 to endogenous hypoxic niches to modulate plant development
Source: New Phytol. 2020 Mar 16;229(1):126–39. doi: 10.1111/nph.16477 (PMC7754370; doi:10.1111/nph.16477)
Supplement: Supplementary file 1 — Fig. S1 Genotyping PCR confirming homozygosity of mutants in the Col‐0 FRI‐Sf2 background. Table S1 Primer sequences used in this study. Please note: Wiley Blackwell are not responsible for the content or functionality of any Supporting Information supplied by the authors. Any queries (other than missing material) should be directed to the New Phytologist Central Office. [file NPH-229-126-s001.pdf]

***New Phytologist* Supporting Information**

Article title: **The PRT6 N-degron pathway restricts VERNALIZATION 2 to endogenous hypoxic niches to modulate plant development**

Authors: Anne-Marie Labandera, Hannah M. Tedds, Mark Bailey, Colleen Sprigg, Ross D. Etherington, Olunwatunmise Akintewe, Geetika Kalleechurn, Michael J. Holdsworth and Daniel J. Gibbs

Article acceptance date: 04 February 2020

The following Supporting Information is available for this article:

Fig. S1 Genotyping details for mutants in the FRI-Sf2 background

Table S1 List of primers used in this study

Fig. S1 Genotyping PCR confirming homozygosity of mutants in the Col-0 FRI-Sf2 background. SDW = water control.

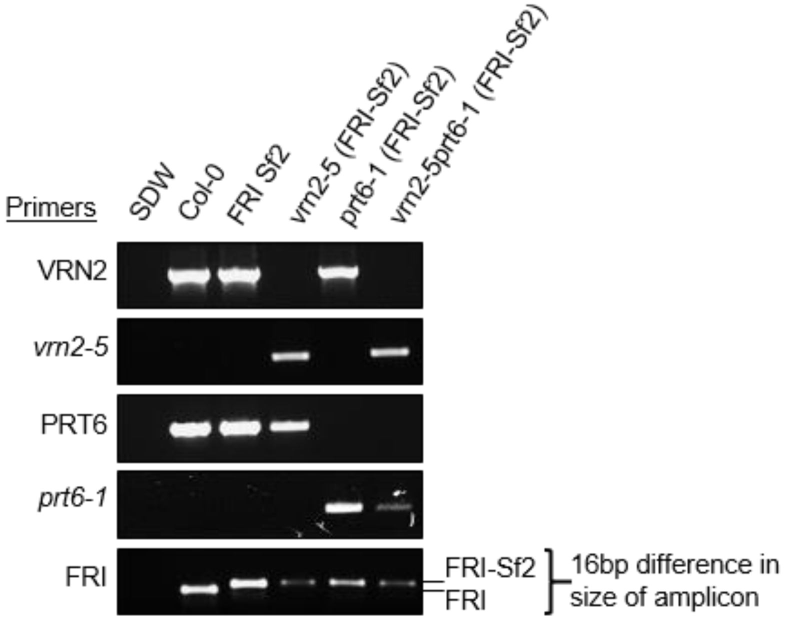

Table S1 List of primers used in this study

Primer name	AGI code	sequence
Primers for RT-PCR		
VRN2 F	At4g16845	ATGTGTAGGCAGAATTGTCGCGC
VRN2 R	At4g16845	TTACTTGTCTCTGCTGTTATTGTCC
GUS fusion R	-	TGGCACAGCAATTGCCCGGC
ACTIN2 F	At3g18780	ATGGCTGAGGCTGATGATATTC
ACTIN2 R	At3g18780	AGAAACATTTTCTGTGAACGATTC
VIN3 F	At5g57380	ATGAAGCTGCTTCGCTCTC
VIN3 R	At5g57380	ATGCCAAAGCTTGAGGCAG
Primers for qPCR		
qPCR FLC F	At5g10140	AGCCAAGAAGACCGAACTCA
qPCR FLC R	At5g10140	TTTGTCCAGCAGGTGACATC
qPCR ADH1 F	At1g77120	GGTCTTGGTGCTGTTGGTTT
qPCR ADH1 R	At1g77120	CTCAGCGATCACCTGTTGAA
qPCR ACTIN2 F	At3g18780	TCGTACAACCGGTATTGTGCTG
qPCR ACTIN2 R	At3g18780	TTACAATTTCCCGCTCTGCTG
qPCR VIN3 F	At5g57380	TGC TTGTGGATCGTCTTGTCA
qPCR VIN3 R	At5g57380	TTCTCCAGCATCCGAGCAAG
qPCR VRN2 P1 F	At4g16845	AATTGGGGCAAAGCGCAAAA
qPCR VRN2 P1 R	At4g16845	AAATTGCAGCCCCTTGAAGC
qPCR VRN2 P2 F	At4g16845	TCACTCTTCTGGTGTTGGAGA
qPCR VRN2 P2 R	At4g16845	GATGGTGGCTGAGTCGACAA
Genotyping primers		
vrn2 F	At4g16845	GTTTGTTCATCATGACCCC
vrn2 R	At4g16845	TTTGAGTCACTGGGATGATCC
SALK LBb1.3	-	ATTTTGCCGATTTCGGAAC
prt6 F	At5g02310	GGAGTTTTCTATGTCCAGTGAGAGTTT
prt6 R	At5g02310	GTCTCCAATGACACGTTCACTTGTCT
prt6 BP	-	GCCTTTTCAGAAATGGATAAATAGCCTTGCTTCC
FRI-Sf2 F	At4g00650	AGATTTGCTGGATTTGATAAGG (Johanson *et al.*, 2000)
FRI-Sf2 R	At4g00650	ATATTTGATGTGCTCTCC (Johanson *et al.*, 2000)

References

Johanson U, West J, Lister C, Michaels S, Amasino R, Dean C. 2000. Molecular analysis of FRIGIDA, a major determinant of natural variation in Arabidopsis flowering time. *Science* **290**(5490): 344-347.
